# Buffalo Pathological Society

**Published:** 1890-06

**Authors:** E. A. Smith

**Affiliations:** Secretary


### BUFFALO PATHOLOGICAL SOCIETY.

Reported by E. A. SMITH, M. D., *Secretary.*

Regular meeting was held Friday, April 18th. The president, Dr. DeLancey Rochester, in the chair.

Dr. M. A. Crockett read a paper on Arteritis, and was followed by Dr. A. W. Hurd, who read on The Symptomatology and Treatment of Arteritis. Dr. C. C. Frederick then read on Phlebitis and Lymphangitis, its Etiology and Pathology.

DISCUSSION.

Dr. Eli H. Long said that the two sets of papers had interested him very much, and had caused him to compare arteritis and phlebitis. Some cases of obscure thrombosis have been ascribed to phlebo-sclerosis of the pulmonary veins. In connection with lymphangitis he called attention to lymphadenitis, this was sometimes seen in perfectly healthy individuals, and it gave rise to palpable lymphatic glands, which were not at all indicative of syphilis or tuberculosis.

Dr. Parmenter was glad that Dr. Long had called attention to the frequent enlargement of the cubital glands, and said that they were often neglected by the profession when examining for syphilis. In cases of epistaxis and acute exacerbations of chronic gastric disease he always looks to the condition of the arteries, for he has found that disease of the arteries often seems to stand in causative relation with these cases.

Dr. Grove said that in his experience cases speedily die which combine persistent headache, epistaxis, and the ophthalmoscope shows a recent retinal clot.

Dr. Krauss mentioned a case where a microscopical examination of a specimen showed a perfect picture of phlebitis, while the accompanying artery was entirely normal.

Dr. C. C. Frederick said he had often observed that in the aged, when there exists a moderate arterio-sclerosis, alcohol was of much benefit. He wondered whether this was due to its effects on the heart, or its dilating effect on the vessels.

Dr. B. G. Long mentioned a case where to all appearances the man was entirely healthy, save a slight gastric trouble. Examination of the urine gave a trace of albumin and a few hyaline casts. At another examination no albumin or casts were found. This man died suddenly eight weeks after the first examination.

Dr. C. C. Frederick mentioned a case of a young man who had been rejected by two insurance orders because of a slight trace of albumin in the urine. If this man was given whiskey in moderate amounts for forty-eight hours no albumin could be found in the urine for a few days after.

Dr. CROCKETT said, closing, that artheroma and calcification of arteries were not synonymous and should not be mixed.

Dr. HURD said that in arterial disease the gastric trouble was often ascribed to hepatic arteritis.

